# Amphiphilic Cellulose Nanocrystals for Aqueous Processing of Thermoplastics

**DOI:** 10.1021/acsapm.2c01623

**Published:** 2022-11-01

**Authors:** Amaka J. Onyianta, Anita Etale, Todor T. Koev, Jean-Charles Eloi, Yaroslav Z. Khimyak, Stephen J. Eichhorn

**Affiliations:** † Bristol Composites Institute, School of Civil, Aerospace and Mechanical Engineering, University of Bristol, BristolBS8 1TR, U.K.; ‡ School of Chemistry, University of Bristol, BristolBS8 1TS, U.K.; § School of Pharmacy, University of East Anglia, Norwich Research Park, NorwichNR4 7TJ, U.K.

**Keywords:** cellulose nanocrystals (CNCs), hydrophobic modification, amphiphilic cellulose, polypropylene, thermoplastic composites, nanocomposites

## Abstract

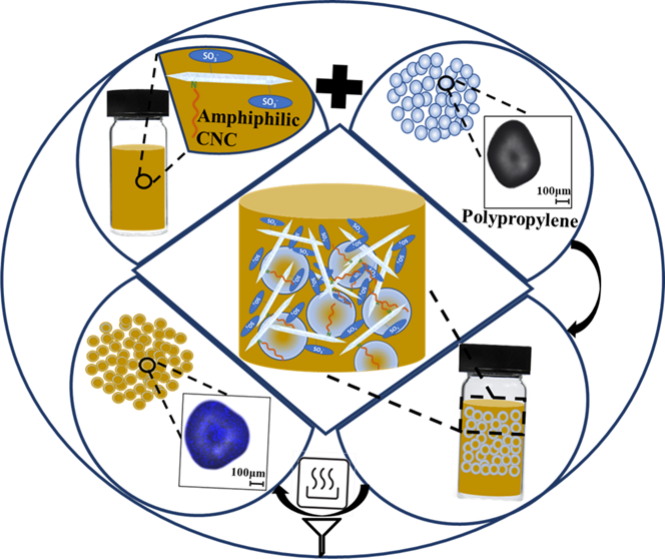

Conventional composite formulation of cellulose nanocrystals (CNCs) with thermoplastics involves melt compounding or in situ polymerisation. In this rather unconventional approach, polypropylene (PP) microparticles were finely suspended and stabilized, at varying weight loadings, in aqueous suspensions of amphiphilic CNCs to enable adsorption of the nanoparticles onto the thermoplastic. In order to achieve these suspensions, CNCs were modified with either octyl or hexadecyl groups. These modifications imparted hydrophobic properties to the CNCs, hence increasing interfacial adhesion to the PP microparticles. The modification, however, also retained the sulfate half ester groups that ensured dispersibility in aqueous media. The CNCs were evidently coated on the PP microparticles as revealed by confocal microscope imaging and had no detrimental effect on the melt properties of the PP-based composites. The approach is demonstrated to increase the Young’s moduli of CNC-thermoplastic composites prepared in optimum suspension loadings of 0.5 wt. % octyl-modified and 0.1 wt % hexadecyl-modified CNCs. This procedure can be extended to other thermoplastics as the ability to aqueously process these composites is a major step forward in the drive for more sustainable manufacturing.

## 1 Introduction

Polymer nanocomposites are generally prepared with the intention of harnessing the individual material properties of the components to prepare a new system with enhanced properties.^1−5^ These improvements in properties are widely claimed to occur when certain material and interfacial requirements are satisfied. The requirements include, but are not limited to, a homogenous dispersion and similar surface energies of the components.^6^ These composites generally include the matrix polymer, fillers, and additives. Natural fibers are increasingly becoming attractive over synthetic fibers because of their lower density and their not being recalcitrant in the environment.^7,8^

Cellulose nanocrystals (CNC) are rod-like colloidal nanoparticles, typically generated by acid hydrolysis of plant biomass,^9,10^ that can act as fillers within various polymer matrices, resulting in mechanically robust composites.^11,12^ The mechanisms of reinforcement are generally described to occur through stress transfer within percolated CNC networks^12^ or stress transfer between the CNC phase and the polymer matrix.^13^ CNCs, just like their cellulosic biomass starting material, are endowed with hydroxyl groups in addition to sulfate half ester groups, when extracted with sulfuric acid.^14,15^ These surface functionalities, however, render them highly hydrophilic with very little inherent hydrophobic properties.^16,17^

The inherent hydrophilicity of CNCs makes them easier to be formulated with other hydrophilic polymers matrices such as polyvinyl alcohol (PVA),^18^ sodium alginates,^19^ and starch^20^ without modification. Cellulose itself has, however, been recently reported to possess amphiphilic properties;^21,22^ however, various modifications and processing methods have been proposed for CNCs to increase dispersion in hydrophobic matrices. These include grafting of hydrophobic moieties such as 6–12 carbon chain alkyl groups,^23,24^ polystyrene,^25^ benzyl-polyethyleneimine,^26^ and *n*-butyl/isobutyl and tertbutyl groups.^27^ These so-called amphiphilic CNCs have been mainly prepared to act as emulsifiers in Pickering emulsions.^24−27^

For CNCs to be effectively incorporated and dispersed in hydrophobic thermoplastics, which are intrinsically incompatible with highly hydrophilic CNCs, similar modifications to increase their hydrophobic properties are required. Without these modifications, aggregation of CNCs occurs when dried CNCs are directly mixed with hydrophobic polymers because of dissimilar surface properties, with the CNCs preferring each other over the polymer matrix.^28,29^ One of these interventions includes liquid phase exchange of aqueous CNCs to acetone, followed by mixing with a hot solution of low density polyethylene.^30^ Other approaches have involved the dispersion of freeze-dried CNCs in dimethyl formamide^31^ or their modification with (2-dodecen-1-yl) succinic anhydride^32^ prior to incorporation into a polyurethane pre-polymer. The mechanical strength of a polypropylene–polyethylene blend was increased with the addition of 2–15 wt % hydrophobized microfibrillated cellulose prepared via modification with tannic acid and coupling with octadecyl (C_18_) groups.^33^ The study, however, still showed aggregation of the hydrophobized cellulose within the composite, which impeded their impact properties.^33^

Composite formulations of thermoplastics with nanocellulose are generally formed through melt compounding of dried nanocellulose and the polymer matrix and in situ polymerization.^34^ A non-conventional method of formulation would be to mix the thermoplastic material in an aqueous dispersion of the nanocellulose that possesses similar interfacial properties, in other words, to form an aqueous emulsion. Interfacial adsorption of amphiphilic nanocellulose has been attempted by Kondo et al.^35^ on isotactic polypropylene (PP) microparticles. The adsorption of a low concentration of cellulose nanofibrils onto the PP microparticles was clearly seen from confocal laser scanning microscopic images and resulted in a depression of the PP melting point.^35^ It was claimed that the inherent amphiphilicity of the nanocellulose enabled their dispersion in aqueous media and adhesion to polypropylene, although it could possibly be due to van der Waals forces.

An efficient way of increasing the amphiphilicity of charged CNCs is through the coupling of different chain lengths of alkyl amines (C_6_, C_8_, and C_12_).^23^ These alkylated CNCs retain a portion of the sulfate half ester groups that ensure stability in aqueous suspensions while showing increases in water contact angle as a result of the alkyl groups. In addition, the aqueous suspensions of alkylated CNCs in potassium chloride formed robust gels with higher storage moduli when compared with those of sulfated CNCs at the same weight loadings.^23^ Octyl-modified CNCs have also recently been used to stabilize linseed oil-water Pickering emulsions for self-healing composite coatings, showing their capacity to interact with hydrophobic materials.^24^ However, studies on the interaction of aqueous suspensions of amphiphilic CNCs with hydrophobic thermoplastics are still lacking.

We herein report the use of octyl and hexadecyl grafted CNCs for aqueous processing of thermoplastics. This approach harnesses CNCs that possess both hydrophilic and hydrophobic properties. These properties enable them to be dispersed in aqueous media due to the presence of surface charge yet appreciably adsorb to the surface of polypropylene microparticles due to the hydrophobic groups. The material properties of the alkylated CNCs are presented and compared with those of the starting sulfated CNCs. The behavior of the CNC-PP dispersions, the evidence of their coating of PP pellets, and the thermal and mechanical properties of the resulting composites are also presented. This approach has real potential to contribute in several ways to the manufacture of composite materials using aqueous processing, harnessing the amphiphilic properties of modified nanocelluloses. There are many applications of this type of processing. For thermoplastic-based composites using nanocellulose to become ubiquitous and readily processable, batch quantities of combined materials will be required. Our approach goes some way to achieving that aim.

## 2 Experimental Section

### 2.1 Materials

Sodium form sulfated CNCs (11.5 wt %) were supplied by the University of Maine. Potassium periodate, sodium chloride, octylamine, hexadecylamine, sodium cyanoborohydride, isopropyl alcohol, calcofluor white, and ion-exchange resin were supplied by Sigma Aldrich/Merck (Dorset, United Kingdom). Isotactic polypropylene (PP) microparticles were kindly provided by Prof T. Kondo of Kyushu University, Japan, but were originally supplied by Prime Polymer Co., Ltd. (Tokyo, Japan). Ultrapure water was used for all experiments.

### 2.2 Modification of Sulfated CNCs with Alkyl Groups and Characterization

The details of the modification process have been previously reported^23,24^ and are presented in more detail in the Supporting Information. Briefly, the modification, schematically represented in Figure S1 (Supporting Information), involves a two-step process starting with oxidation of sulfated CNCs with potassium periodate to form dialdehyde CNCs.^36,37^ This is followed by the coupling of octylamine or hexadecylamine to dialdehyde CNCs via a reductive amination route. The CNCs are thereafter denoted as sCNCs (sulfated CNCs), oCNCs (octyl CNCs), and hexdCNC (hexadecyl CNCs).

A greater understanding of these alkylated CNCs were pursued through material characterization and comparisons with the starting materials.

Conductometric titration was carried out to quantify the total surface charge of the CNCs emanating from the sulfate half ester groups. For this experiment, all CNC suspensions were protonated for 1 h using an ion-exchange resin. 0.05 g solid weight equivalent of the CNCs were each dispersed in a known volume of deionized water, stabilized with 0.01 M NaCl and stirred for at least 30 min before titrating with 0.048 M of standardized NaOH. Duplicate experiments were carried out for each sample and the average total surface charge reported.

Zeta potential measurements were conducted on CNC samples that were dispersed in 5 mM NaCl solution using Zetasizer Nano-ZS (Malvern, UK) with a DTS 1070 capillary cell. Measurements were carried out at the refractive index of water (*n* = 1.33) and at a temperature of 25 °C. Measurements were carried out in triplicate per sample, and 60 runs were undertaken per measurement.

Fourier transform infrared (FTIR) spectroscopy was used to analyze the changes in the surface functionalities using a Spectrum 100 spectrometer (Perkin-Elmer, USA) equipped with a diamond crystal ATR tip. Freeze-dried samples were used to obtain the spectra from 4000 to 600 cm^–1^ at a resolution of 4 cm^–1^.

Solid-state NMR experiments were performed on a Bruker Avance III NMR spectrometer, equipped with a 4 mm triple resonance probe operating at frequencies of 300.13 MHz (^1^H) and 75.48 MHz (^13^C). CNC powder samples were packed tightly into an 80 μL rotor and spun at a MAS rate of 12 kHz. All ^1^H–^13^C CP/MAS NMR spectra were acquired at 20 °C using 12 k scans, a recycle delay of 10 s, and a contact time of 2 ms. Spectral deconvolution was performed via a global spectral deconvolution algorithm using the MestreLab MNova (v14.2) software package.

Degree of surface modification was calculated according to eq 1.^23,24,38^
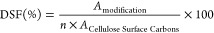
1where *A*_modification_ is the area under the deconvoluted peaks of the aliphatic moieties (octyl and hexadecyl), *n* is the number of carbon atoms in the aliphatic decoration (8 for octyl and 16 for hexadecyl), and *A*_Cellulose Surface Carbons_ is the sum of the area under the deconvoluted peaks of the surface C-4 and C-6 atoms (sC4 and sC6, respectively; Figure S2 of Supporting Information).

Transmission electron microscopy (TEM) was used to image the changes in the CNCs’ morphology at 200 kV in the bright field mode, on a JEOL JEM-2100F microscope equipped with an Orius SC1000 CCD camera from Gatan. A suspension (0.001 wt %) of never-dried CNCs was sonicated at a 10% amplitude for 10 min and deposited onto freshly glow-discharged carbon-coated copper grids, to increase their surface hydrophilicity. The specimens were subsequently negatively stained with aqueous 2 wt % uranyl acetate before drying and imaging.

Contact angle analysis was carried out to assess changes in the hydrophobicity of CNCs as a result of alkyl chain grafting*.* For this, 1 wt % solutions of surface-modified- and -unmodified CNCs were cast onto glass slides and left to dry at ambient temperature. A drop (10 μL) of deionized water was then dropped onto the films, and digital images of the profiles of the droplets were recorded every 2 s for 60 s. All measurements were performed in duplicate, under ambient conditions.

Thermal gravimetric analysis (TGA) was carried out to assess the thermal stabilities of the CNCs upon modification with alkylamines. Sodium and acid forms of freeze-dried CNCs were investigated. TGA was carried out using Netzsch simultaneous thermal analyzer, STA 449 (Netzsch, Germany). 15–20 mg of each sample was heated in an aluminum oxide pan from 30 to 600 °C at a heating rate of 10 °C min^–1^ under a constant nitrogen flow of 50 mL min^–1^. DTG curves were obtained by performing a first derivative on the % weight loss data from TGA using the Origin 2020b software package.

### 2.3 CNC-PP Composite Formation by Surface Adsorption

**Figure 1:**
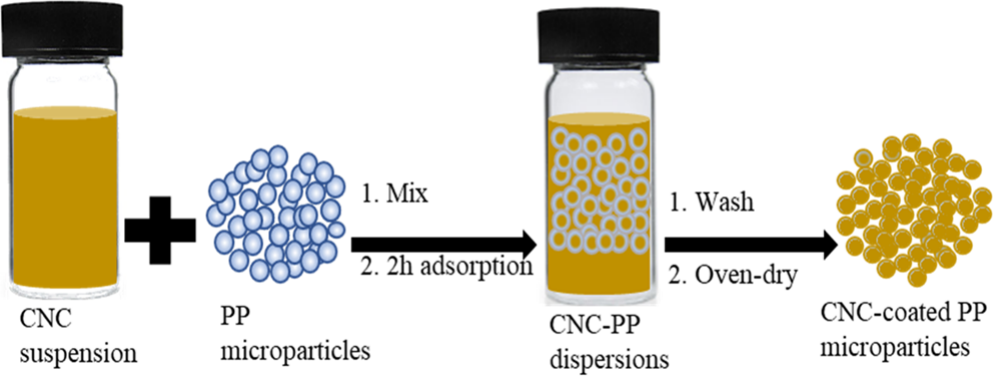
Schematic representation of the adsorption process of CNCs onto PP microparticles.

CNC-PP composites were formed through surface adsorptions of the aqueous suspensions of the CNCs on the PP microparticles. The method used for the adsorption experiments was adapted from Ishikawa et al. (2021).^35^ 0.1, 0.5, and 1 wt % aqueous suspensions were prepared from stock suspensions of sCNC, oCNC, and hexdCNC in glass vials. The required amounts of PP microparticles were added to each vial at 15 wt % and shaken vigorously before allowing them to stand for 2 h. The PP microparticles were then filtered off and washed with ultrapure water to remove unbound CNCs. The PP microparticles with adsorbed CNCs were then dried in the oven for a minimum of 1.5 h at 105 °C. The adsorption process is schematically represented in Figure 1. The dried CNC-PP composite microparticles were directly molded to desired shapes using an injection molding instrument.

### 2.4 Characterization of CNC-PP Composites

The interactions between the CNCs and PP microparticles were studied through visual assessments of the composite suspensions. Also, the dried composite microparticles as well as the cross sections of the injection molded composites were studied with confocal laser scanning microscope (CLSM) imaging. The CNC-adsorbed PP microparticles were stained with calcofluor white, a fluorescent stain for cellulose, to examine the coating of CNCs on the PP microparticles. 0.4 mL of calcofluor white was added to 0.1 g of PP in a 1.5 mL Eppendorf tube. The mixture was shaken, and the staining process was allowed to take place for 30 min. The calcofluor white stain was removed, and the PP microparticles were washed with ultrapure water and dried at 50 °C for 3 h. Z-stack images were generated using T10 CLSM (Leica Microsystems, Wetzlar, Germany) at an excitation wavelength of 405 nm.^45,46^

Scanning electron microscope (SEM) images and energy-dispersive X-ray (EDX) spectra were collected from the surfaces of the PP and a representative CNC-PP composite microparticle. For this, a JSM-IT300 scanning electron microscope from JEOL Japan at 15 kV was used. The samples were coated with Ag at a 45° angle to ensure a conductive coating from the top surface to the stage. Micrographs were taken at a working distance of 10 mm. EDX data were collected using an X-Max 80 mm^2^ EDX detector and analyzed with AZtec software, both from Oxford Instruments, UK.

To examine the distribution of the CNCs within the molded composites, cross sections were stained with calcofluor white, washed thoroughly with water, and air-dried. Confocal laser scanning microscopic images were captured using a 10× magnification lens on the T10 microscope and at an excitation wavelength of 405 nm.

Differential scanning calorimetry (DSC) analyses were carried out on the CNC-PP composite microparticles to examine whether the addition of the nanomaterial had any effect on the bulk melting profile of PP. DSC was carried out using Netzsch DSC 204 Phoenix (Netzsch, Germany). 8–10 mg of PP microparticles or its composites with the 3 CNC types were hermetically sealed in a Concavus aluminum pan with a pierced lid and heated to 200 °C at a heating rate of 10 °C min^–1^ under a constant nitrogen flow of 40 mL min^–1^.

Tensile testing was performed on the dumbbell-shaped samples at a speed of 1 mm min^–1^ using a 10 kN load cell on a Shimadzu AGX tensile testing instrument (Shimadzu, Kyoto, Japan). To make the dumbbell specimens, PP microparticles and their composites with CNCs were molded according to ASTM D 638 Type V using a Haake Pro Piston Minijet (Thermo Fisher Scientific, UK) injection molding instrument. During tensile testing, the strain values were measured using a video extensometer (IMETRUM, Bristol, UK). A minimum of 5 dumbbells were tested per sample. Young’s modulus was obtained as the initial slope of the true stress–strain curve. The tensile strengths were reported for each sample as the maximum stress sustained by the sample, also known as the ultimate tensile strength.

Statistical analyses of tensile test results were carried out. Each loading of the different CNCs was compared to that of the uncoated PP as well as between the composites using a two-sample *t*-test using Origin 2020b software. The difference between means was considered significant when *p* ≤ 0.05, and an equal variance was assumed.

## 3 Results and Discussion

### 3.1 Functional Group Analyses by Conductometric Titration, Zeta Potential, FTIR Spectroscopy, and ^1^H–^13^C CP/MAS NMR

Conductometric titration showed that the total surface charges of the sCNC, oCNC, and hexdCNC were 336.1 ± 0.4, 236.8 ± 1.9, and 77.5 ± 1.9 mmol kg^–1^, respectively. The values obtained for sCNC and oCNC are of the same order with those previously reported by Nigmatullin et al.^23^ It can be seen from the results that increasing the carbon chain length led to a systematic decrease in the overall surface charge. This downward trend is also observed from zeta potential measurements, with results showing charge potentials of −36.5 ± 0.2, −33.2 ± 1.1, and −28.2 ± 0.6 for sCNC, oCNC, and hexdCNC, respectively.

Theoretically, the amount of sulfate half ester groups on the C6 carbon, which is measured by titration, is not expected to be affected by the modification process. Therefore, the surface groups should remain unchanged. This is based on the understanding that the reductive amination reaction is occurring at the aldehyde groups on the C2 and C3 carbons of the cellulose chains.^39,40^ However, the reduction that is observed herein could result from the obstruction of the sulfate half ester groups on adjacent cellulose chains within the CNC chains by the alkyl groups. Similarly, a reduction in aldehyde content was observed for polystyrene-modified CNCs, which was also attributed to a charge screening effect by the hydrophobic moiety.^25^

**Figure 2:**
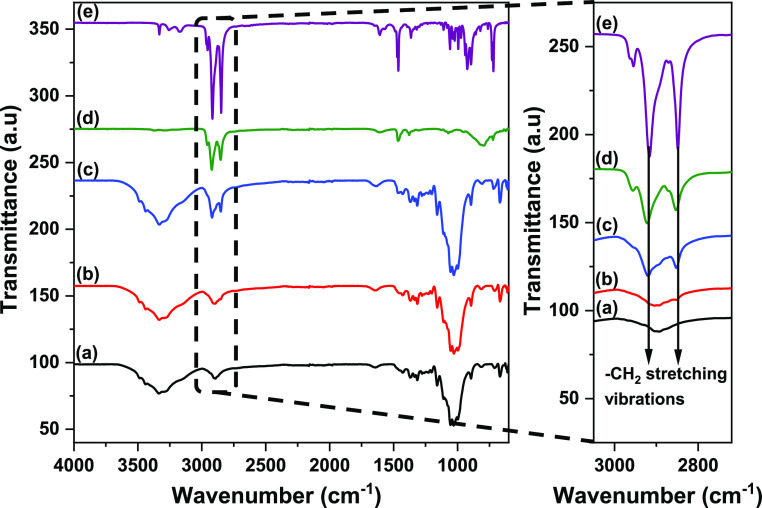
Typical FTIR spectra of sCNCs (a), oCNCs (b), and hexdCNCs (c) and those of the alkylamines: octylamine (d) and hexadecyl amine (e). The spectral region showing −CH_2_ stretching from alkyl groups are magnified and shown on the right.

FTIR spectroscopy was conducted to examine if there were any identifiable changes in the functional groups of the sulfated CNCs, before and after modification with alkylamine. Figure 2 shows that the infrared spectra of the sCNCs, oCNCs, and hexdCNCs have spectral bands typically observed for cellulose. These characteristic bands are for the O–H (∼3322 cm^–1^), C–H (∼2899 cm^–1^), and C–O (∼1027 cm^–1^) stretching vibrations.

In the spectra for oCNC and hexdCNC, a shoulder to the band located at ∼2852 cm^–1^ is noted, which is also observed for pure octylamine and hexadecylamine. This band is relatively small for the oCNC sample and is assigned to the asymmetrical stretching vibration of −CH_2_ of the long chain alkyl groups.^39,40^ The symmetrical −CH_2_ stretching vibrations located at ∼2923 cm^–1^ were identified for hexdCNCs and both alkylamines but not for oCNCs. These −CH_2_ bands were not present in the spectra for sCNCs, which is a strong indication that successful modifications with long chain alkyl groups occurred, albeit to a low degree for oCNC.

**Figure 3:**
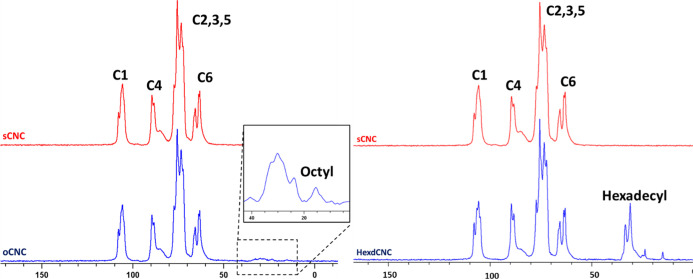
Typical ^1^H–^13^C CP/MAS NMR spectral overlay of sCNC with oCNC and hexdCNC, with the inlay showing the zoomed spectral region of the octyl moiety (left).

The ^1^H–^13^C CP/MAS NMR spectra shown in Figure 3, of sCNC, oCNC, and hexdCNC, demonstrated the presence of octyl and hexadecyl moieties on the CNC surface.^25^ The degree of surface functionalization (DSF) of oCNCs was 2.3%, in agreement with previous works.^23,24^

The DSF of hexdCNC was found to be 9.7%, which is more than 4-fold higher than that of oCNC. This considerable increase in the DSF of hexdCNC compared to that of oCNC may help explain the drop in surface charge, likely because of the bulky hexadecyl moieties obstructing the sulfate groups on neighboring CNC chains.^23−25^

### 3.2 Effects of Surface Modification on the Morphology, Contact Angle, and Thermal Stabilities of Alkylated CNCs

**Figure 4:**
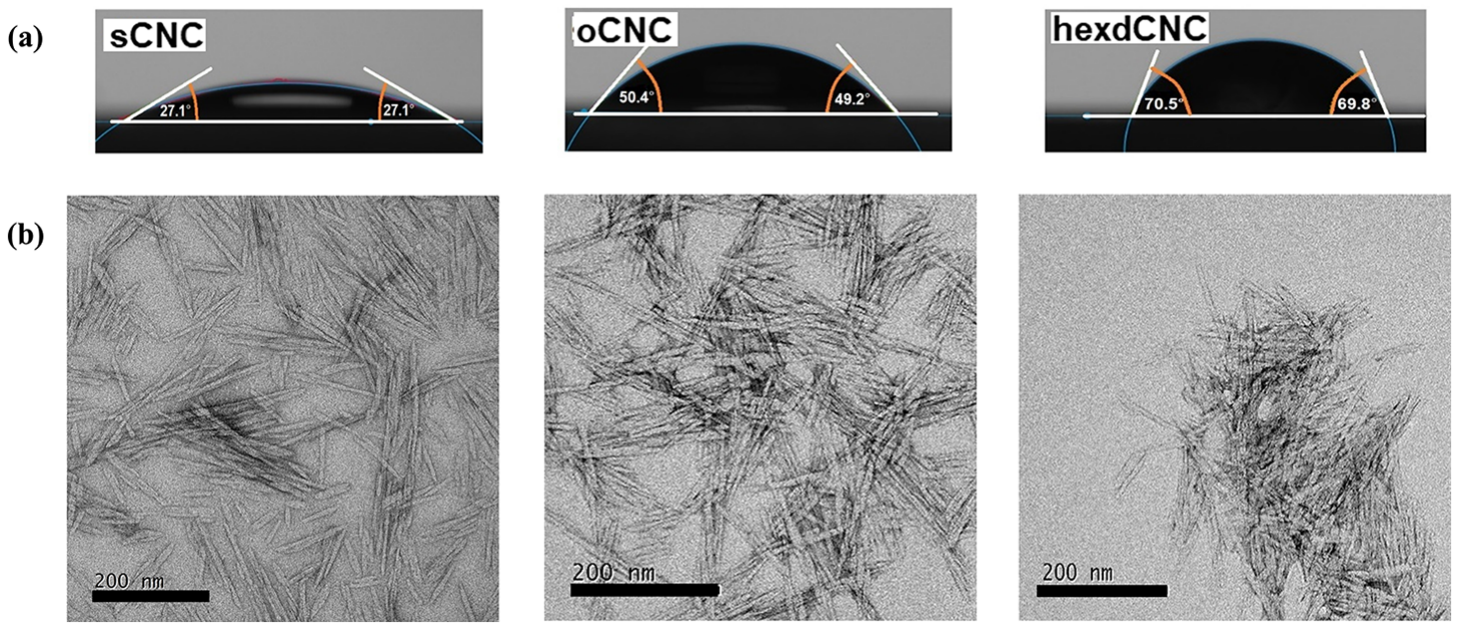
Typical contact angles of water droplets on sCNCs, oCNCs, and hexdCNCs (a) and TEM images of sCNCs, oCNCs, and hexdCNCs (b, left to right); images show increasing degree of aggregation with increasing carbon chain length on the CNCs. Scale bar = 200 nm.

The results of the contact angle measurements Figure 4a showed that the hexdCNC surface was more hydrophobic than the oCNC surface, that is, 70 and 50° contact angles, respectively. The sCNC samples, as expected, were hydrophilic (27°). Modification of the CNCs with octylamine and hexadecylamine therefore increased the hydrophobicity of sCNCs to a degree that was in tandem with the polymer chain length; hexdCNCs were more hydrophobic than oCNCs. Importantly, the hydrophobicity values attained for both oCNCs and hexdCNCs suggest that some hydrophilicity was retained within the CNCs and that the materials were not transformed to a superhydrophobic state. We suggest that this is likely responsible for the interaction of CNC-coated PP microparticles with water as we discuss later.

Representative TEM images of sCNC, oCNC, and hexdCNC are presented in Figure 4b. oCNC and hexdCNC maintained rod-like features as the starting sCNCs.^41^ Aggregation seems to increase with increasing alkyl chain length. However, it is far more pronounced for hexdCNC, an observation which supports the initial assumption that the alkyl groups are shielding the sulfate half ester groups. Similar crystal aggregation was also observed for CNCs modified with hydrophobic polystyrene.^25^

**Figure 5:**
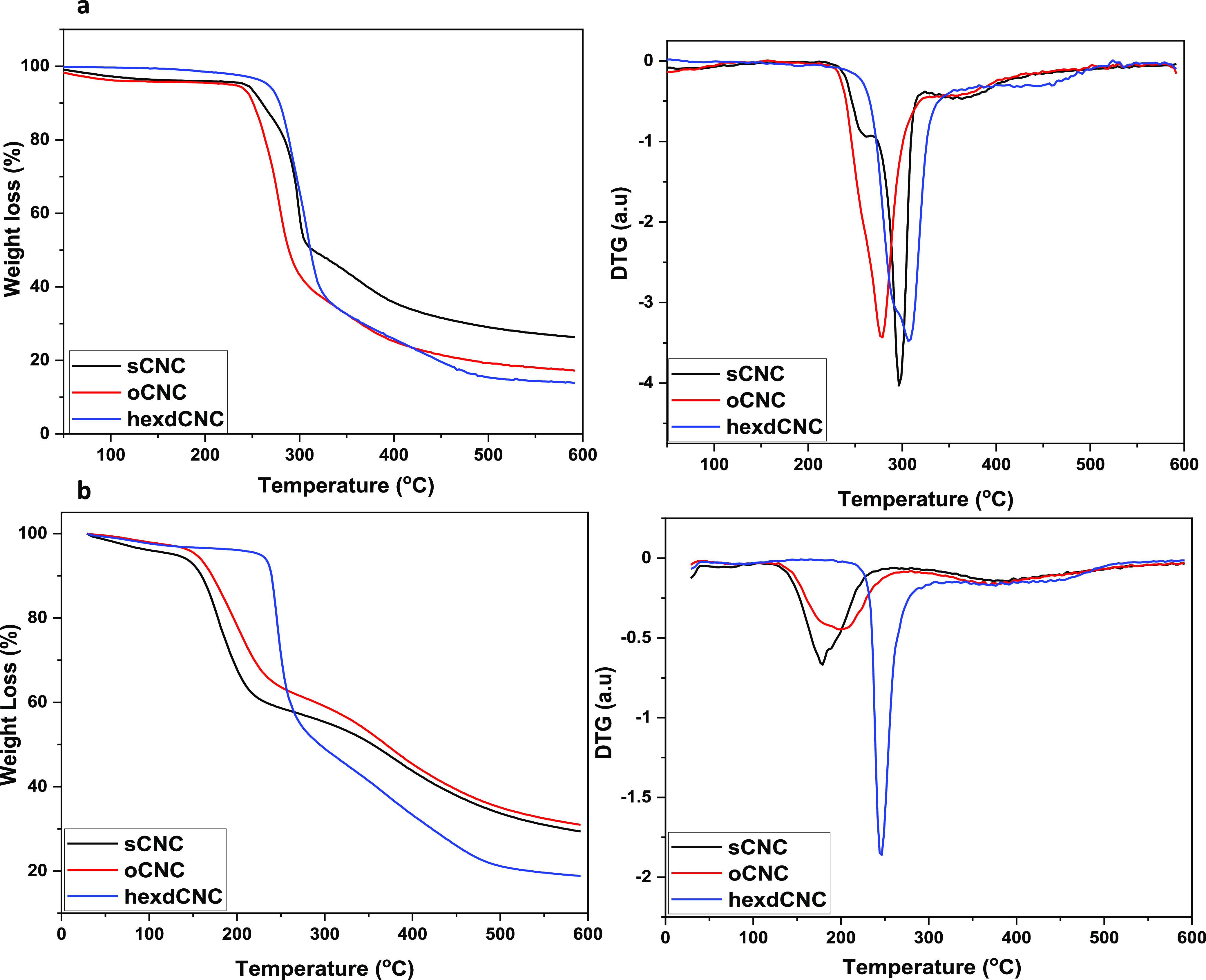
TGA and DTG thermograms of sodium form (a) and acid form (b) sCNCs, oCNCs, and hexdCNCs showing the differences in thermal stabilities of the different materials.

The effects of the alkyl amine modification of sulfated CNCs on their thermal profile were studied by performing a dynamic heating ramp on the sodium form and acid form freeze-dried materials up to 600 °C. The thermograms and their derivative curves are presented in Figures 5a and 5b. For the sodium form CNCs (Figure 5a), the respective onset degradation temperatures of sCNC, oCNC, and hexdCNC are ∼247, ∼245, and ∼272 °C. From the DTG curves, however, two peak degradation temperatures can be identified for sCNC (at ∼259 and ∼297 °C). The initial peak degradation temperature identified for sulfated CNCs is attributed to the degradation of the thermally unstable sodium half sulfate ester groups.^42^ No initial degradation temperature was seen for oCNC and hexdCNC, but single peak degradation temperatures of ∼278 and ∼308 °C were observed. It is noted that there is no clear trend on the effect of the alkyl chain length on the thermal properties of sodium form CNCs when compared with the sulfated CNC. To understand the effect of the modification without the impact of the sodium ion, which has been reported to improve the thermal stabilities of sulfated CNCs,^42,43^ protonated CNCs were also thermally examined (Figure 5b).

The impact of sodium ions on the thermal stability of the sCNCs was very apparent, with the onset and peak degradation temperatures reducing to ∼147 and ∼179 °C, respectively. A clearer trend in the impact of the modification on the thermal stabilities of the CNCs could be seen for oCNCs and hexdCNCs. As has been previously postulated, the presence of the alkyl groups shields the sodium half ester groups from being exchanged to the acid form, thereby shifting the onset and peak degradation of oCNC to slightly higher temperatures of ∼152 and ∼202 °C. This level of increase in comparison to sCNCs is in line with the degree of functionalization of oCNC. Likewise, hexdCNC, having a higher degree of functionalization and larger shielding of the sodium half ester groups, resulted in a more thermally stable amphiphilic CNC with a higher onset and peak degradation temperatures of ∼231 and ∼245 °C, respectively. The relationship between total surface charge and peak degradation temperature is graphically represented in Figure S3 of the Supporting Information.

### 3.3 Aqueous Suspensions of PP Microparticles and CNCs

The alkyl group modified CNCs were found to disperse well in water. However, hexdCNCs appeared to sediment at lower weight loadings, an obvious effect of the lower surface charge and aggregation discussed previously. Rheological analyses of the alkylated CNCs, in 2 wt % aqueous suspensions, show that the storage modulus of oCNC is 60% higher than hexdCNC (Supporting Information, Figure S4).

**Figure 6:**
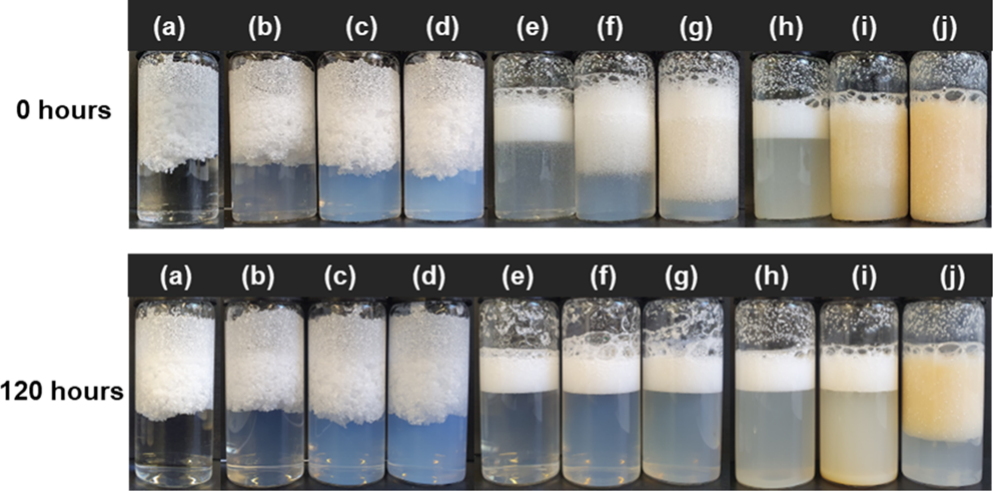
Dispersions of PP in water (a) and PP in different weight loadings of alkyl CNC suspensions: 0.1 wt % sCNC (b), 0.5 wt % sCNC (c), 1 wt % sCNC (d), 0.1 wt % oCNC (e), 0.5 wt % oCNC (f), 1 wt % oCNC (g), 0.1 wt % hexdCNC (h), 0.5 wt % hexdCNC (i), and 1 wt % hexdCNC (j).

Photographs of PP in water and in various aqueous suspensions of sCNC, oCNC, and hexdCNC are presented in Figure 6. They show that PP microparticles did not mix with water or sCNC at all weight loadings; their separation from these mixtures after mixing was rapid. Furthermore, on close inspection, it was clear that PP particles in these two systems coalesced into lumps, which is likely due to dissimilar surface properties of the PP and water/sCNC.

In contrast, oCNC-coated PP microparticles did not coalesce to the same extent and were more dispersed in the suspension. Also, the separation of particles from the water decreased with an increasing loading of the oCNC suspension. The fine dispersions obtained at all weight loadings are an indication of the better interaction between the octyl groups on oCNC and PP microparticles. This increased interaction, which is intensified at higher weight loadings of oCNC, may also be responsible for the lower rate of separation of coated PP microparticles at 0.5 and 1 wt.% oCNC.^22^

**Figure 7:**
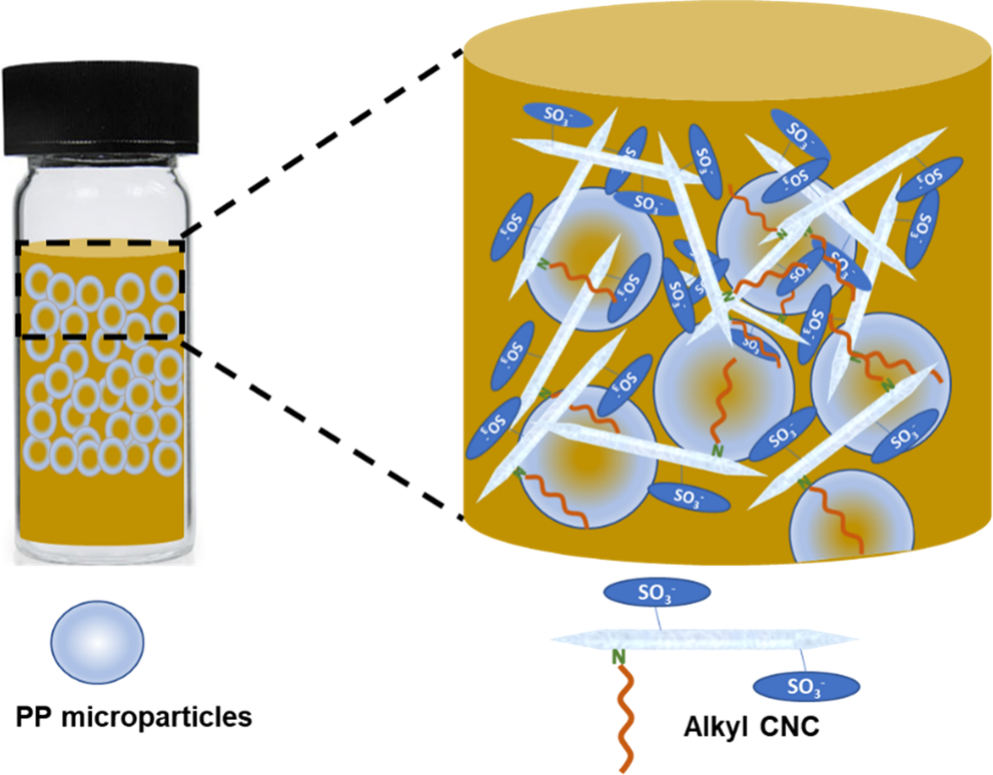
Stabilization of PP microparticles in aqueous suspensions of alkyl CNCs. Here, CNC acts as an emulsifier with negatively charged sites, stabilizing the nanocrystals in water and hydrophobic ends binding to PP.

As represented in Figure 7, the behavior observed here can be likened to the Pickering emulsion system where oCNC functions as an emulsifier, having both negatively charged sites and hydrophobic surfaces that stabilize the oil droplets (in this case, PP microparticles) in water.^24^

The surface properties of the particles and the fluid, as well as the fluid rheology, are two of the factors that can affect the movement of particles in a suspension.^44^ Therefore, it is possible that the surface properties and higher storage modulus of oCNC could have contributed to the ability of the PP microparticles to suspend in the aqueous media. However, for hexdCNC suspensions which displayed lower storage moduli, the hydrophobic effects of 0.5 and 1 wt % were greater and may have superseded the fluid rheology, thereby also finely stabilizing the PP microparticles in the aqueous suspension. In fact, at 1 wt.% hexdCNC, no separation of PP microparticles could be observed within the first hour.

After 120 h of standing, the PP particles in all oCNC suspensions, alongside those in 0.1 and 0.5 wt % hexdCNC suspensions, floated to the top of the mixture. The 1 wt % hexdCNC–PP mixture, after 120 h, however, showed a higher “float height” as can be seen in Figure 6. It can, therefore, be said that increasing the hydrophobicity of sCNC by modification with octylamine and hexadecylamine increased the interaction of the CNC and PP and resulted in coated materials with enhanced dispersion and stabilization in water.

### 3.4 Confocal Laser Scanning Microscopic (CLSM) and Scanning Electron Microscopic Image Analyses of the Adsorption of CNCs on PP Microparticles

Staining of the CNC-PP composites with a fluorescent calcofluor white dye facilitates the visualization of the affinity of the different CNCs, at various weight loadings, to the PP surfaces. An increase in the intensity of the stain would indicate greater adsorption and affinity of the alkyl CNCs to the PP microparticles.

**Figure 8:**
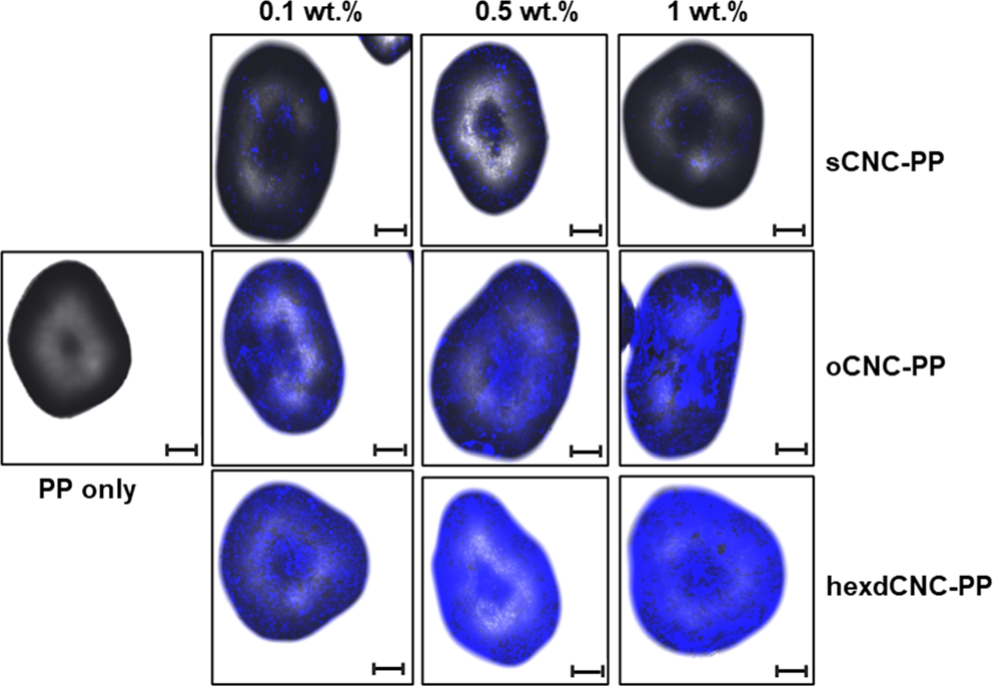
Typical CLSM images of neat PP microparticles and those coated with sCNCs, oCNCs, and hexdCNCs at 0.1, 0.5, and 1 wt % loadings of CNC suspensions. The length of the scale bar is 100 μm.

Figure 8 shows the confocal laser scanning micrographs of uncoated as well as sCNC-, oCNC-, and hexdCNC-coated PP at different weight loadings. The uncoated PP microparticles show no fluorescence, but varying degrees of staining can be seen on the surfaces of sCNC-coated PP particles, even though no trend in the intensity of the stain was observed with the increasing loading of sCNCs. Sulfated CNCs are thought to be a predominantly hydrophilic material, and adsorption on the surface of the PP microparticles is expected to be minimal. Yet the presence of some fluorescence suggests that some degree of coating of the PP surface by these largely hydrophilic materials has occurred. Some have argued that these sort of interactions could be because cellulose is amphiphilic,^22,35^ but it is likely that van der Waals forces play a significant role in all interactions between PP and CNCs. Similar findings were reported for interactions between cellulose nanofibrils, prepared through the aqueous counter collision process, with PP microparticles.^35^

Nevertheless, oCNCs and hexdCNCs clearly have a much greater affinity for PP microparticles, to a much greater extent than sCNCs. Further, a clear trend is observed between the intensity of fluorescence and the loading of the oCNC from 0.1 to 1 wt %. PP microparticles coated with lower oCNC loading showed an even distribution of the nanomaterial on the surfaces.

However, at the highest loading investigated (1 wt %), uneven areas of fluorescence suggest some level of aggregation and therefore an uneven distribution of alkyl CNCs on the PP surface. The hexdCNCs clearly exhibit the greatest intensity of the fluorescent stain in comparison to sCNC- and oCNC-coated PP. This suggests an increased affinity to the PP surface with the increasing alkyl chain length, which ultimately also leads to a greater stabilization of hexdCNC-coated PP particles, as these were more hydrophobic as a result of the longer alkyl chains. A similar trend is also seen for the effect of the weight loading on the intensity of the stain; the intensity increased as the weight loading of the hexdCNCs increased from 0.1 to 1 wt %, even though the difference in fluorescence intensity between 0.5 and 1 wt % hexdCNC PP particles is not marked. Nevertheless, particles coated in a 0.1 wt. % hexdCNC suspension appear to have a better distribution of the CNCs on the surface of the PP microparticles, which is likely due to less aggregation.

On further investigation of the microstructure of the PP and CNC-PP composite microparticles with SEM/EDX, it could be seen from Figure S5a of the Supporting Information that the surface of neat PP is rough and has negligible oxygen as would be expected of polypropylene. However, when dispersed in amphiphilic CNCs (represented with 1 wt % hexdCNC in Figure S5b), the surface of PP was coated with a layer/film of CNC. This is evidenced by the appearance of a strong oxygen signal in the EDX spectrum (Figure S5b Spectrum 1). There appears to be parts of the microsphere which are not well coated or may have been peeled off during the washing/drying and handling processes (Figure S5b, Spectrum 2).

**Figure 9:**
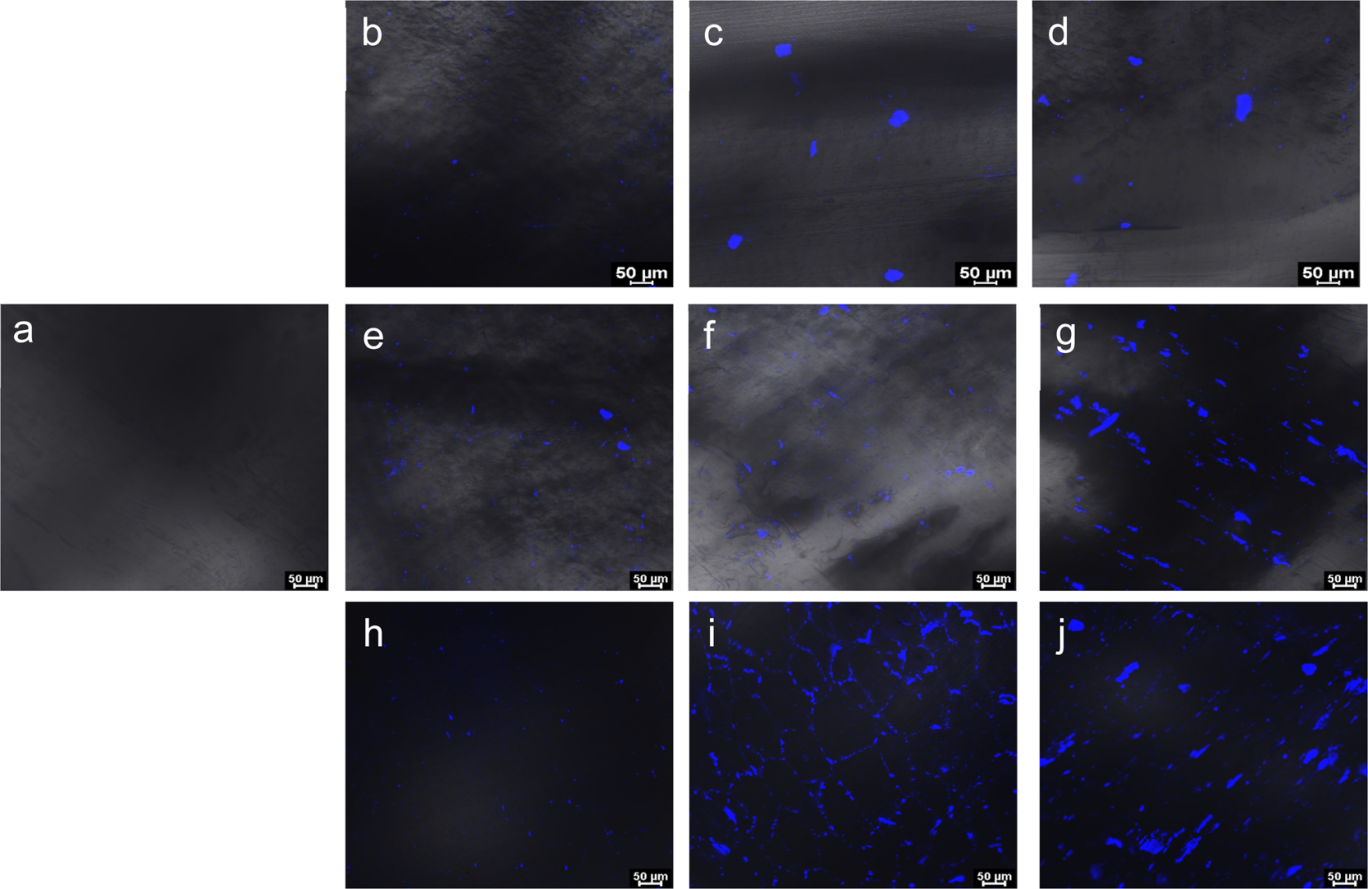
Confocal laser scanning microscopic images of cross sections of neat PP (a); 0.1, 0.5, and 1 wt % sCNC (b, c, and d); 0.1, 0.5, and 1 wt % oCNC (e, f, and g); and 0.1, 0.5, and 1 wt % hexdCNC (h, i, and j). The blue color indicates the location of the CNCs.

During the injection molding process, the coated surfaces are expected to coalesce on melting the PP composites. Therefore, depending on the level of coating on the surface, determined by the loadings of the CNCs in suspension, this may be advantageous or detrimental to the properties of the composites. In order to understand the distribution of the CNCs in the composites, cross sections of neat PP and those of the composites were studied with CLSM and are presented in Figure 9. The blue particulate materials represent the fluorescing CNCs, which are not present in the neat polypropylene cross section. Across the three groups of CNCs investigated in this study, composites dispersed in lower loadings of CNCs (0.1 wt %) yielded better distribution of the CNCs. Increased coverage of the PP with higher loadings of CNCs is manifested with the presence of larger CNC particles. This trend is more pronounced for the PP composites prepared from alkylated CNCs than for sulfated CNCs.

### 3.5 Melting Profiles and Mechanical Properties of PP Composites with sCNCs and Alkyl Modified CNCs

The DSC thermograms of the PP and CNC-PP composites are shown in Figure S6. The addition of the sCNC, oCNC, or hexdCNC at different weight loadings did not meaningfully affect the peak melting temperatures of PP composites. It is noted that, although the peak thermal degradation temperatures of oCNCs were lower than those of sCNCs and hexdCNCs, the addition of the CNCs to the PP microparticles did not adversely affect the melting profile of PP.

**Figure 10:**
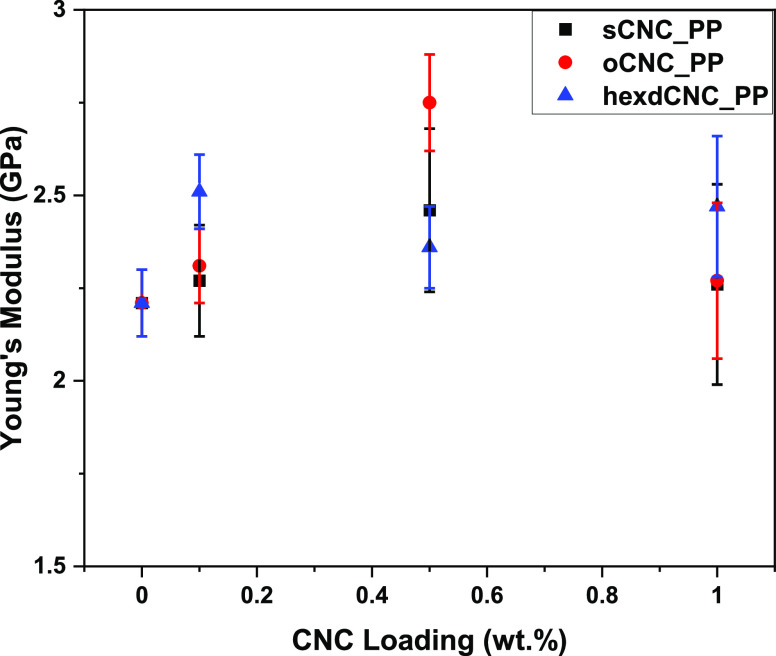
Effect of the different CNC materials at different % weight loadings on the mechanical properties of the CNC-PP composites.

The mechanical properties of the CNC-PP composites were studied using tensile testing. Young’s moduli are presented in Figure 10 and in Table 1 alongside the tensile strengths. Full stress/strain curves are presented in Figure S7 of the Supporting Information. Uncoated PP microparticles are represented in Figure 10 as a 0 wt % CNC loading. According to the statistical analyses performed between the PP and the composites prepared in varying loadings of CNCs, significant increases of ∼24 and ∼14% were observed when PP microparticles were dispersed in 0.5 wt % oCNC and 0.1 wt % hexdCNC suspensions, respectively, in comparison with the neat PP. Composites prepared from sCNC suspensions or higher loadings of oCNC and hexdCNC did not show a statistically significant improvement in the Young’s modulus.

**Table 1:** Young’s Modulus and Tensile Strength Data of PP Composites with sCNC, oCNC, and hexdCNC at Different Weight Loadings

	sCNC-PP		oCNC-PP		hexdCNC-PP	
CNC loading (wt %)	Young’s modulus (GPa)	Tensile strength (MPa)	Young’s modulus (GPa)	Tensile strength (MPa)	Young’s modulus (GPa)	Tensile strength (MPa)
0 (PP only)	2.21 ± 0.09	35.4 ± 0.7	2.21 ± 0.09	35.4 ± 0.7	2.21 ± 0.09	35.4 ± 0.7
0.1	2.27 ± 0.15	34.7 ± 0.4	2.31 ± 0.1	36.0 ± 0.2	2.52 ± 0.10	34.8 ± 0.9
0.5	2.46 ± 0.22	35.6 ± 0.3	2.75 ± 0.13	35.8 ± 0.4	2.36 ± 0.11	34.1 ± 0.8
1	2.26 ± 0.27	35.0 ± 1.6	2.27 ± 0.21	34.9 ± 0.4	2.47 ± 0.19	33.4 ± 0.8

Also, there were no significant changes in tensile strengths of PP composites with sCNCs and oCNCs. However, a significant decrease in tensile strength was observed for composites dispersed in 0.5 and 1 wt % hexdCNC suspensions. For nanoparticles such as CNCs to offer the expected stress transfer and subsequent reinforcement, the particles need to possess similar surface properties and be homogenously dispersed within the matrix.^6^ Aggregation of the nanofiller, which could be seen as highly saturated patches and larger particle sizes in CLSM images (Figures 8 and 9), may have led to the negative effect on the mechanical properties of the composites at higher CNC weight loadings. While the increase in stiffness is noted, decreases in strength may have something to do with the presence of aggregates above the critical flaw size, leading to premature fracture; this type of behavior has been seen before for nanocellulose-based thermoplastic composites.^33^

## Conclusions

The surface modification of cellulose nanocrystals with 8 and 16 carbon chain lengths of alkyl groups has been presented. The amphiphilicity of the alkylated CNCs have been demonstrated through various surface chemistry and physical analyses. The octyl and hexadecyl modified CNCs behaved differently in comparison to the starting material, showing lower surface charge, increasing water contact angle, and increasing storage moduli with increasing carbon chain length. PP microparticles were then finely dispersed in aqueous dispersions of these alkylated CNCs at different weight loadings. This resulted in the coating of the microparticles by the CNCs and increases in Young’s modulus. This study presents a process for composite formulation of thermoplastics with CNCs by aqueous processing. Modulation of the surface properties of CNCs for preferential adsorption to hydrophobic matrices in aqueous media is a desirable approach for future sustainable composites. This approach enables this in a green and readily scalable way. The approach could also be extended to other particulate systems and the CNCs used for the suspending and dispersion of non-charged particles during processing, both organic and inorganic.

## Supporting Information Available

Methodology for modification of CNC with alkylamines; additional NMR spectra; surface charge and peak degradation temperatures; frequency sweep of modified CNC solutions; methodology for viscoelastic measurement of the alkyl CNCs; scanning electron micrographs and EDX spectra of coated PP particles; DSC thermograms of PP composites; and stress–strain curves of PP and PP composites (PDF)
