# Supplementary material for: Amphiphilic Cellulose Nanocrystals for Aqueous Processing of Thermoplastics
Source: ACS Appl Polym Mater. 2022 Nov 1;4(11):8684–93. doi: 10.1021/acsapm.2c01623 (PMC9667462; doi:10.1021/acsapm.2c01623)
Supplement: Supplementary file 1 — ap2c01623_si_001.pdf [file ap2c01623_si_001.pdf]

Supporting Information

Amphiphilic cellulose nanocrystals for aqueous processing of thermoplastics

*Amaka J. Onyianta[1], Anita Etale[1], Todor T. Koev[3], Jean-Charles Eloi[2], Yaroslav Z. Khimyak[3]., Stephen J. Eichhorn[1]**

1.Bristol Composites Institute, Faculty of Civil, Aerospace and Mechanical Engineering, University of Bristol, Bristol, BS8 1TR, UK

2.School of Chemistry, University of Bristol, Bristol, BS8 1TS, UK

3.School of Pharmacy, University of East Anglia, Norwich Research Park, Norwich, NR4 7TJ, UK.

*Corresponding author

Stephen J. Eichhorn: s.j.eichhorn@bristol.ac.uk

Modification of CNC with alkylamines

The modification of CNCs with alkylamine involves a dialdehyde and reductive amination processes, which was carried out herein according to the method previously presented by Nigmatullin et al.[1] and described below.

Public

Dialdehyde modification: To modify the surface of the CNCs with the aldehyde groups needed for an onward reductive amination reaction with alkylamine, periodate modification was first carried out. 44.5 g of 11.5 wt.% CNC (5 g dry weight) was diluted up to 200 mL with water. The dispersion was shaken vigorously and sonicated for 1 min at a 10% amplitude. The dissolution of 1.8 g of KIO_4 was added to the CNC dispersion and stirred for 48 hours at room temperature in a dark environment.

The reaction was quenched after 48 hours by adding 0.8 g of ethylene glycol. Then 250 g of 2 wt.% NaCl was added to the reaction to cause the CNCs to precipitate. The CNCs were recovered by centrifugation and washed 2 more times with 2 wt.% NaCl. The precipitated CNC diluted with water and homogenised for 2 mins at 10000 rpm using an ultra turrax disperser. The suspension was dialysed using dialysis tubing. This was then dialysed against deionised water overnight with frequent changes of water.

Reductive amination with alkylamine: The suspension was divided into 2 portions, and each made up to 200 g with water in a 250 round bottom flask and heated up to 45 °C. Then 5g of octylamine or 4.7g of hexadecylamine were respectively added to the suspension and maintained at 45 °C for 3 hours. After 3 hours, the suspension was cooled to room temperature before adding 1 g of sodium cyanoborohydride and allowed to induce the reductive amination for 21 hours. The modified CNCs were then recovered by centrifugation and washed three times with a 2 wt.% NaCl/IPA (50/50 vol%) mixture before being dialysed for 1 week with frequent changes of water.

The CNC materials were henceforth designated as sCNCs (sulfated CNCs), oCNCs (octyl CNCs) and hexdCNCs (hexadecyl CNCs). The schematic for the two-step modification process is presented in Figure S1.

Public

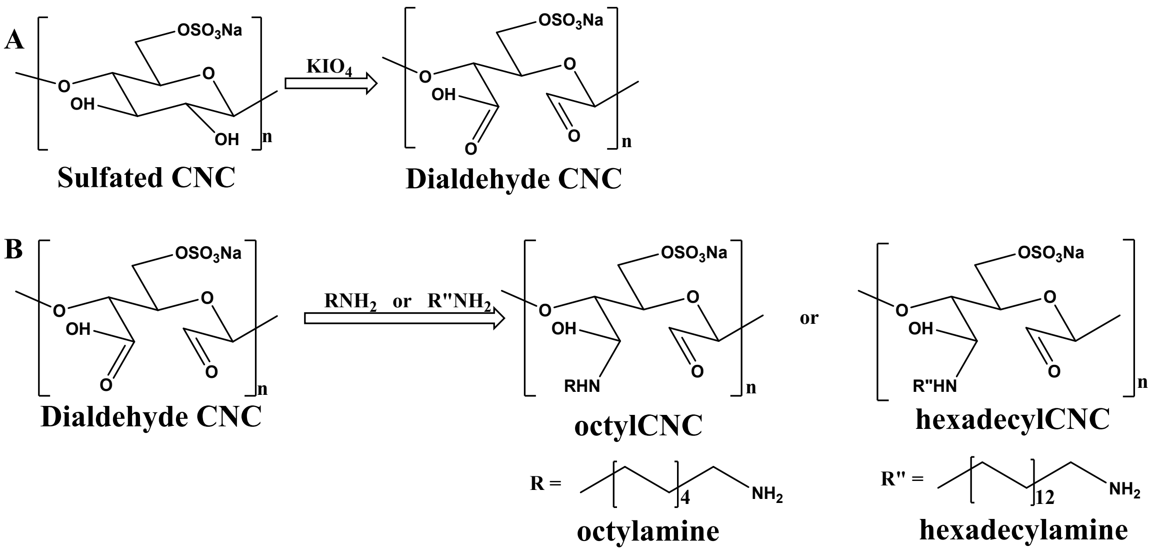

Figure S1: Dialdehyde CNC (A) and reductive amination modification processes with alkylamines

^{1}H–^{13}C CP/MAS NMR spectroscopy

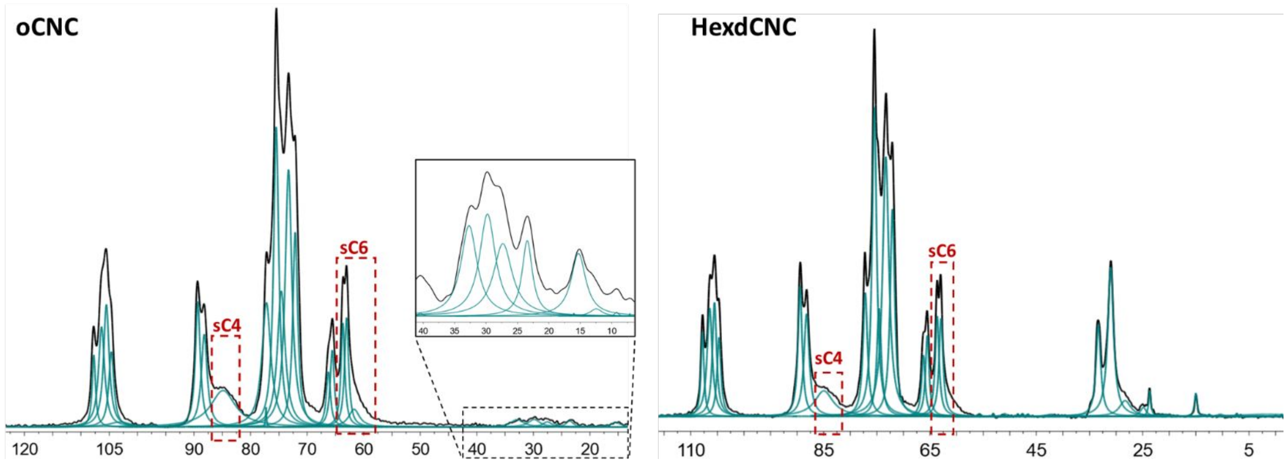

Figure S2: Deconvoluted 1H-13C CP/MAS NMR spectra of oCNC and hexdCNC (left and right, respectively), with the surface C-4 and C-6 peaks shown in red broken line (sC4 and sC6, respectively).

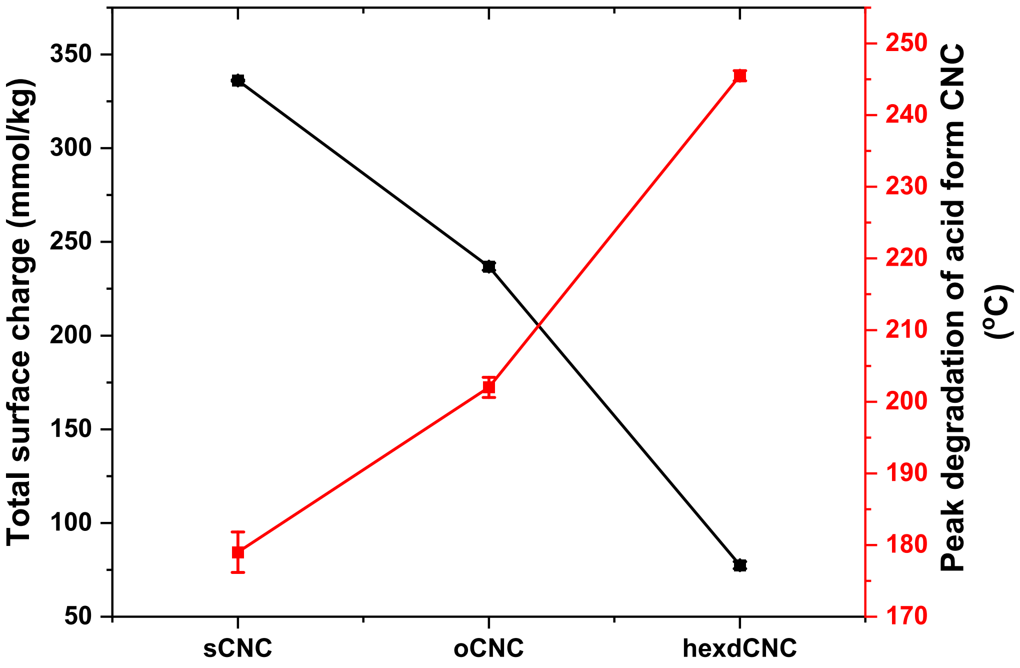

Figure S3: Relationship of total surface charge and peak degradation temperatures of sCNC, oCNC and hexdCNC

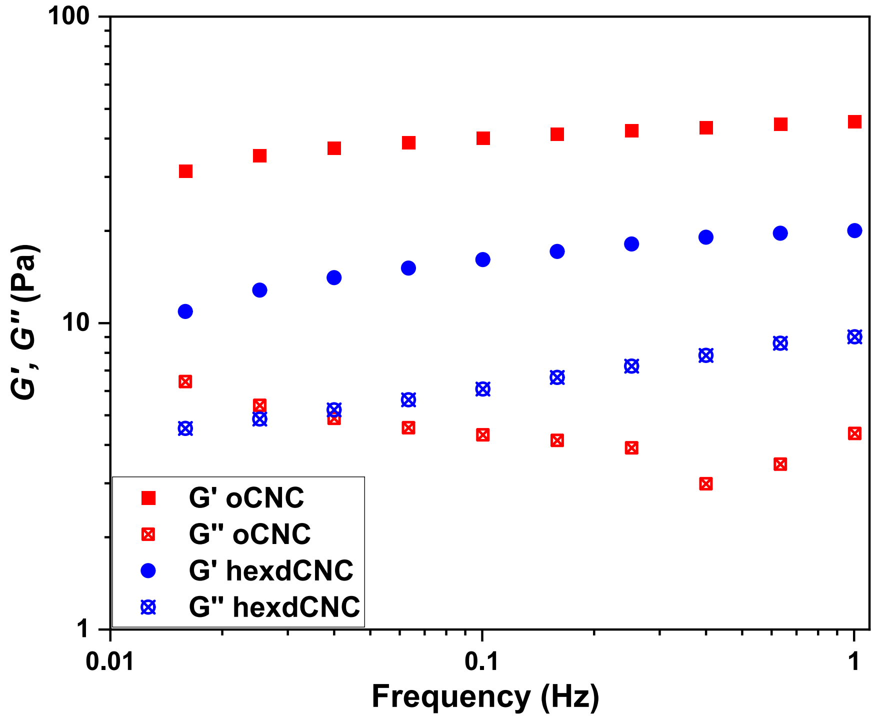

Figure S4: Typical Frequency sweeps of 2 wt.% oCNC (octyl CNC) and hexdCNC (hexadecyl CNC) showing storage (*G*') and loss (*G*'') moduli as a function of angular frequency.

Viscoelastic measurement of the alkyl CNCs

The storage and loss moduli of the alkyl CNCs were measured at 2 wt.% *via* a frequency sweep from 0.015-1 Hz, at a strain value of 0.02 %, using a 40 mm serrated Peltier parallel plate geometry on a DHR rheometer (TA Instruments, USA). Experiments were performed in duplicates and the average data reported. The viscoelastic properties of 2 wt.% alkyl chain modified CNCs were tested, and the results presented in Figure S4. Meaningful data were not obtained for sCNC due to their high viscosity.

The storage modulus (*G*') of oCNC suspension at 1 rads^{-1} is approximately 60 % higher than that of hexdCNC. In comparison with sCNC tested in KCl solution, higher storage modulus has been previously reported for oCNC in the same salt solution.[1] It is known that the storage modulus response observed from colloidal materials is the sum of the electrostatic repulsive forces on the surface of the crystals and any physical entanglements.[4] oCNC largely retains its sulfate half ester group, hence its electrostatic repulsive forces, while at the same time experiencing some increased interaction between the crystals

Public

because of the presence of the octyl groups, hence the high rheological response. For hexdCNC, shielding of the sulfate half ester groups by the longer chain hexdCNC decreased stability and reduced the electrostatic contribution, hence the lower storage modulus.

Public

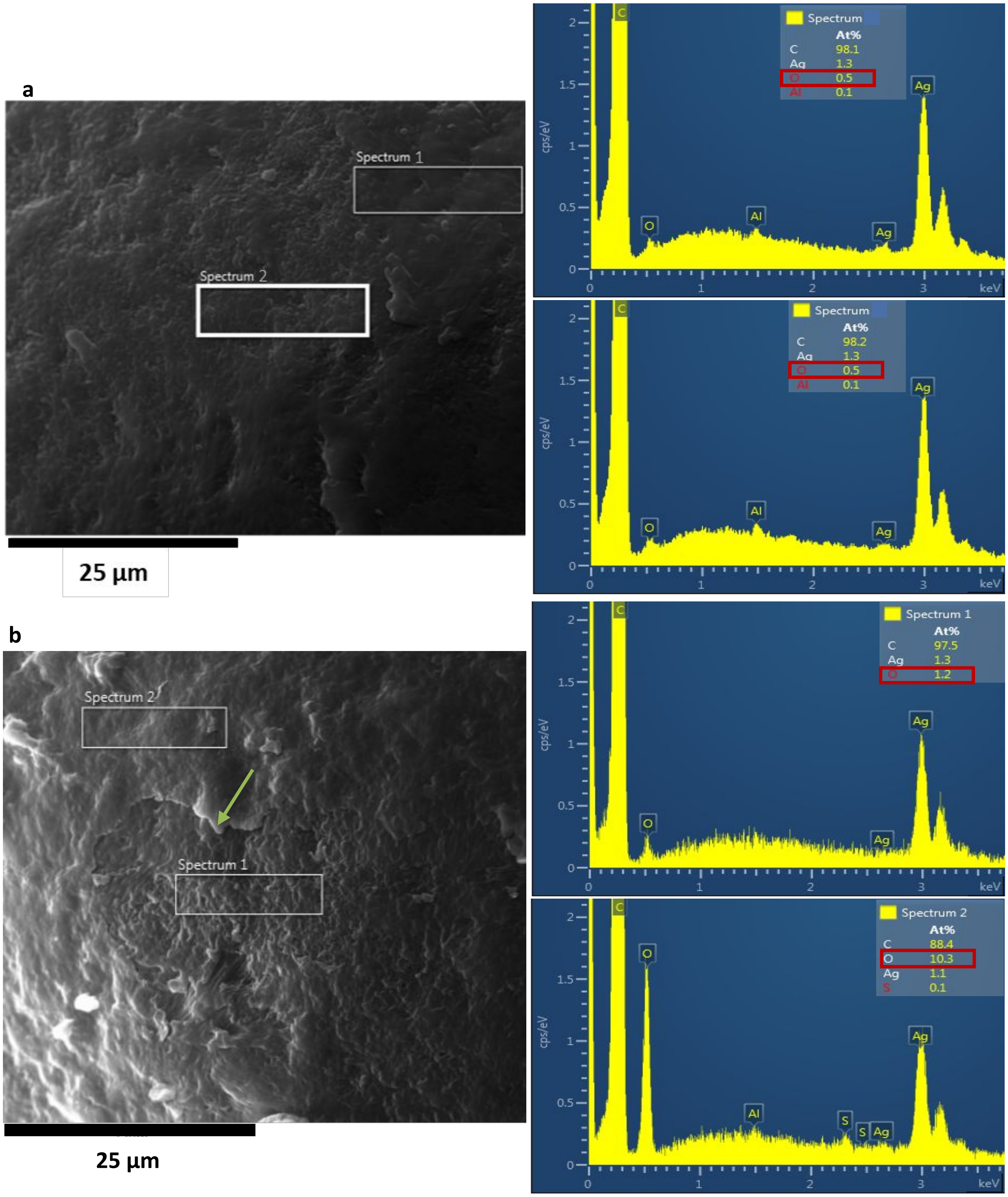

Figure S5: Scanning electron micrographs and EDX spectra of neat PP (a) and PP coated in 1 wt.% hexdCNC (b). The EDX spectra from PP surfaces show negligible amounts of oxygen whereas surfaces coated with hexdCNC (spectrum 2 of 1 wt.% hexdCNC-PP) shows an oxygen rich spectrum. The arrow shows thin CNC film coating.

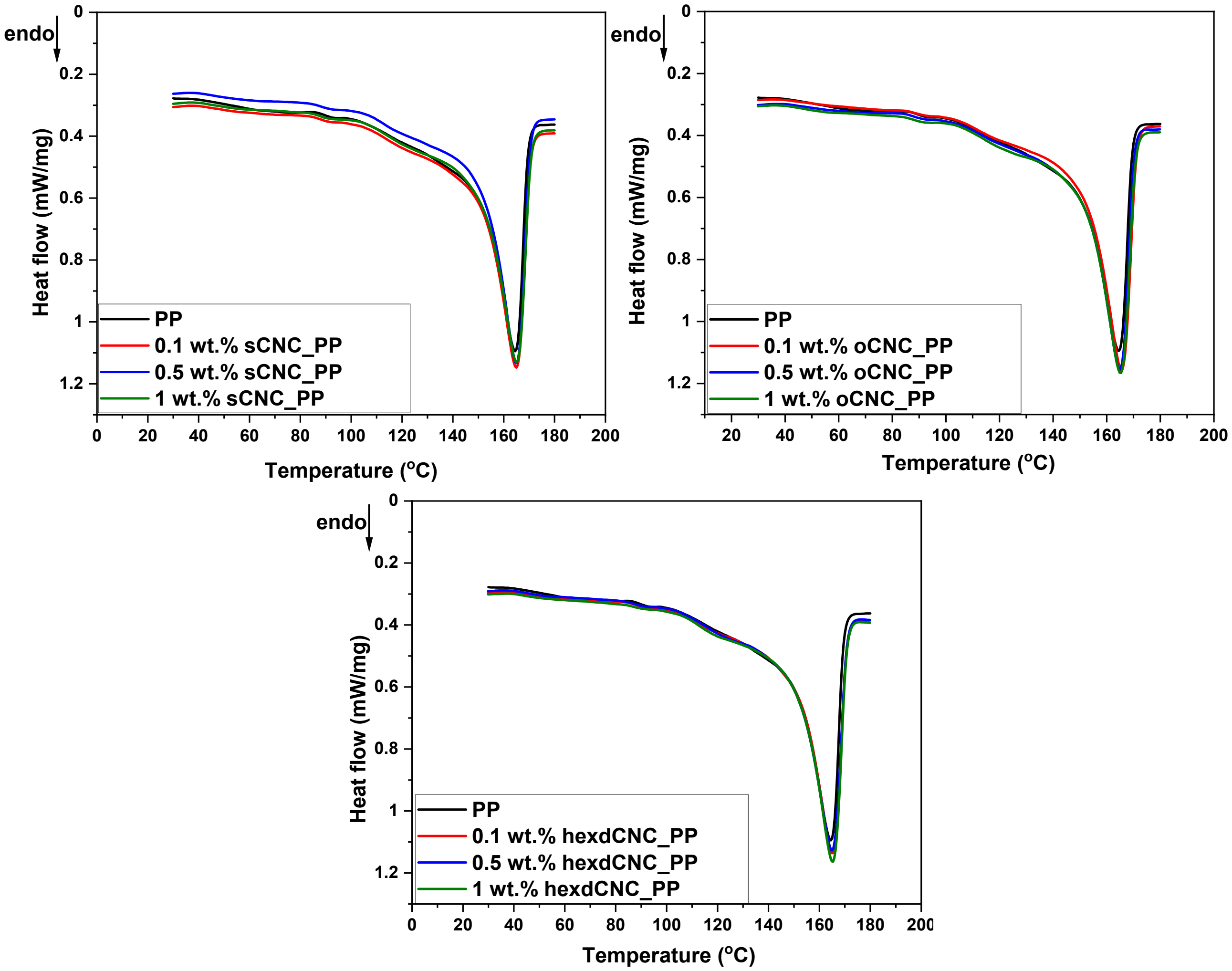

Figure S6: Typical DSC thermograms of composites of PP with sCNCs, oCNCs and hexdCNCs at different weight loadings.

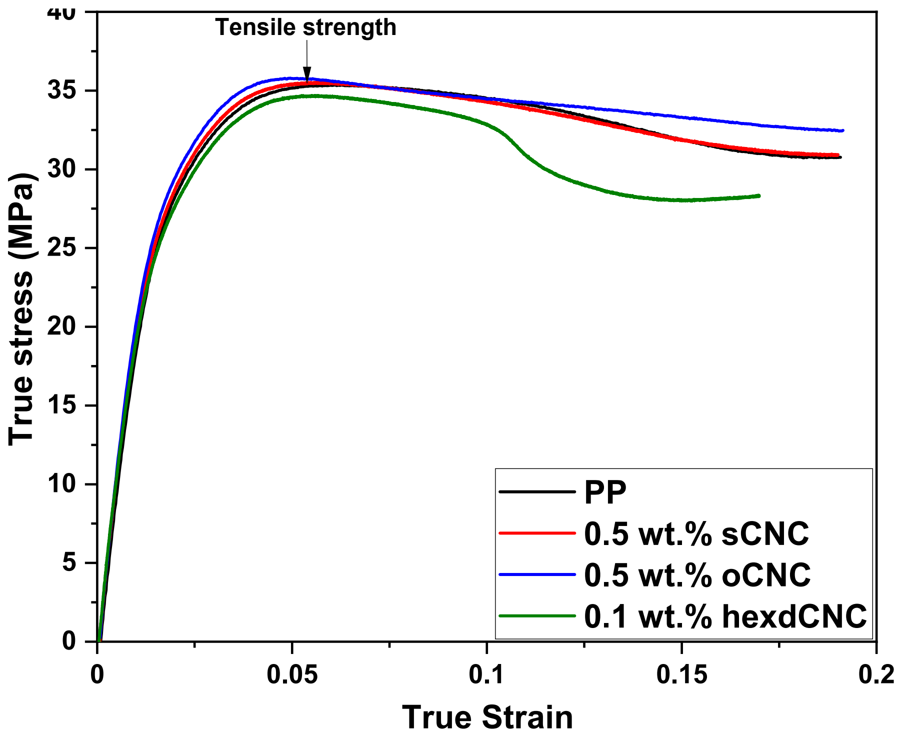

Figure S7: Stress/strain curves of PP and PP composites with sCNC, oCNC and hexdCNC

References

(1) Nigmatullin, R.; Harniman, R.; Gabrielli, V.; Muñoz-García, J. C.; Khimyak, Y. Z.; Angulo, J.; Eichhorn, S. J. Mechanically Robust Gels Formed from Hydrophobized Cellulose Nanocrystals. *ACS Applied Materials and Interfaces* **2018**, *10* (23), 19318–19322. https://doi.org/10.1021/ACSAMI.8B05067/SUPPL_FILE/AM8B05067_SI_001.PDF.

(2) Nigmatullin, R.; Johns, M. A.; Muñoz-García, J. C.; Gabrielli, V.; Schmitt, J.; Angulo, J.; Khimyak, Y. Z.; Scott, J. L.; Edler, K. J.; Eichhorn, S. J. Hydrophobization of Cellulose Nanocrystals for Aqueous Colloidal Suspensions and Gels. *Biomacromolecules* **2020**, *21* (5), 1812–1823. https://doi.org/10.1021/ACS.BIOMAC.9B01721/SUPPL_FILE/BM9B01721_SI_001.PDF.

(3) Xu, G.; Nigmatullin, R.; Koev, T. T.; Khimyak, Y. Z.; Bond, I. P.; Eichhorn, S. J. Octylamine-Modified Cellulose Nanocrystal-Enhanced Stabilization of Pickering Emulsions for Self-Healing Composite Coatings. *ACS Applied Materials and Interfaces* **2022**, *14* (10), 12722–12733. https://doi.org/10.1021/ACSAMI.2C01324/SUPPL_FILE/AM2C01324_SI_001.PDF.

(4) Xu, Y.; Atrens, A. D.; Stokes, J. R. Rheology and Microstructure of Aqueous Suspensions of Nanocrystalline Cellulose Rods. *Journal of Colloid and Interface Science* **2017**, *496*, 130–140. https://doi.org/10.1016/J.JCIS.2017.02.020.
